# A recombinant O-polysaccharide-protein conjugate approach to develop highly specific monoclonal antibodies to Shiga toxin-producing *Escherichia coli* O157 and O145 serogroups

**DOI:** 10.1371/journal.pone.0182452

**Published:** 2017-10-05

**Authors:** Daniela S. Castillo, Diego A. Rey Serantes, Luciano J. Melli, Andrés E. Ciocchini, Juan E. Ugalde, Diego J. Comerci, Alejandro Cassola

**Affiliations:** Instituto de Investigaciones Biotecnológicas - Instituto Tecnológico de Chascomús (IIB-INTECH), Universidad Nacional de San Martín (UNSAM) - Consejo Nacional de Investigaciones Científicas y Técnicas (CONICET), San Martín, Buenos Aires, Argentina; New York Medical College, UNITED STATES

## Abstract

Shiga toxin-producing *Escherichia coli* (STEC) is the major etiologic agent of hemolytic-uremic syndrome (HUS). The high rate of HUS emphasizes the urgency for the implementation of primary prevention strategies to reduce its public health impact. Argentina shows the highest rate of HUS worldwide, being *E*. *coli* O157 the predominant STEC-associated HUS serogroup (>70%), followed by *E*. *coli* O145 (>9%). To specifically detect these serogroups we aimed at developing highly specific monoclonal antibodies (mAbs) against the O-polysaccharide (O-PS) section of the lipopolysaccharide (LPS) of the dominant STEC-associated HUS serogroups in Argentina. The development of hybridomas secreting mAbs against O157 or O145 was carried out through a combined immunization strategy, involving adjuvated-bacterial immunizations followed by immunizations with recombinant O-PS-protein conjugates. We selected hybridoma clones that specifically recognized the engineered O-PS-protein conjugates of O157 or O145 serogroups. Indirect ELISA of heat-killed bacteria showed specific binding to O157 or O145 serogroups, respectively, while no cross-reactivity with other epidemiological important STEC strains, *Brucella abortus*, *Salmonella* group N or *Yersinia enterocolitica* O9 was observed. Western blot analysis showed specific recognition of the sought O-PS section of the LPS by all mAbs. Finally, the ability of the developed mAbs to bind the surface of whole bacteria cells was confirmed by flow cytometry, confocal microscopy and agglutination assays, indicating that these mAbs present an exceptional degree of specificity and relative affinity in the detection and identification of *E*. *coli* O157 and O145 serogroups. These mAbs may be of significant value for clinical diagnosis and food quality control applications. Thus, engineered O-PS specific moieties contained in the recombinant glycoconjugates used for combined immunization and hybridoma selection are an invaluable resource for the development of highly specific mAbs.

## Introduction

Shiga toxin-producing *Escherichia coli* (STEC) pathovar is associated with sporadic cases and outbreaks of diarrhea, bloody diarrhea (BD) and hemolytic-uremic syndrome (HUS), a systemic illness defined by the clinical triad of microangiopathic hemolytic anemia, thrombocytopenia and acute renal failure [1, 2]. STEC strains are characterized by the production of the cytotoxins Shiga toxin 1 (Stx1) or Shiga toxin 2 (Stx2), which have a pivotal role in BD and HUS pathogenesis [3, 4]. Ruminants such as cattle are major reservoirs for pathogenic STEC and exposure to their fecal matter is considered the most frequent source of human illness. Contaminated food and water as well as contact with infected animals or people also represent potential sources of STEC [5–8]. *E*. *coli* O157:H7 is the predominant etiologic serotype of sporadic and outbreak-related BD and HUS illnesses worldwide [4]. However, six other serogroups −O145, O121, O111, O103, O45 and O26−, also known as the "big six", are associated with a similar disease and their prevalence differs geographically [9, 10]. Argentina has the highest global incidence of HUS in children aged 5 years or under −12 to 14 cases per 100,000 children annually− and, with Australia and Germany, is considered a worldwide "hot spot" where non-O157 STEC serogroups are an increasing cause of HUS [10–13]. In Argentina, the dominant STEC-associated HUS serogroup is O157 (>70%), followed by O145 (>9%) and O121 (>2%) [11]. Postdiarrheal HUS in Argentina is endemic and the leading cause of acute renal dysfunction among children [14]. The mortality rate in acute phase is 2 to 5%, but since 20 to 30% of HUS-affected children develop long-term renal sequelae, HUS constitutes the second cause of chronic renal failure and accounts for 20% of kidney transplants in children and adolescents [11, 15–18].

Currently, there are limited prevention strategies for the development of HUS following STEC infection. There is no effective clinical treatment and most patients recover with supportive care, yet 30% of them are left with long-term renal or neurological impairment [19]. Given the high rate of HUS and the lack of specific treatment and high morbidity, primary prevention of STEC infections is essential to reduce its public health impact. For this reason, accurate and rapid technologies are needed to monitor the levels of pathogenic STEC in cattle and food manufacturing facilities. One of the most important risk factors for STEC infection is the consumption of raw or undercooked meat. In Argentina, the meat consumption per person is 60 kg/year. Moreover, 20% of the children in this country start consuming meat at 5 months old, reaching a consumption rate of three times a week at 8 months of age [16].

Bacterial glycoengineering −a discipline that merges the knowledge from bacterial glycobiology and genetic engineering− has emerged in the last years as an advantageous alternative to produce recombinant glycoproteins useful for therapeutics, vaccines and as antigens for diagnosis [20–23]. The N-glycosylation machinery of *Campylobacter jejuni* is the most thoroughly studied bacterial glycosylation system. It has been shown that *C*. *jejuni* N-oligosaccharyltransferase (OTase) PglB, due to its relaxed substrate specificity, is able to transfer a range of different LPS O-PSs from its lipid donor to carrier proteins in a system that combines the N-glycosylation system of *C*. *jejuni* with the O-PS biosynthesis pathway of Gram-negative bacteria. In this *in vivo* bacterial system, the O-PS linked to the lipid carrier undecaprenolphosphate is synthesized at the cytoplasmic face of the inner membrane, flipped to the periplasm, polymerized and transferred by PglB to a carrier protein resulting in the synthesis of the O-PS-protein conjugate [24–27]. Therefore, the aforementioned bacterial glycosylation system is a convenient toolbox for engineering a panel of novel and diverse serogroup-specific O-PS-protein conjugates which can be purified from cultures of non-pathogenic bacteria. O-PS-conjugates can be used as antigens that are recognized with high specificity by sera of infected patients with STEC [23], and also by sera of *Brucella* infected cattle [22] and porcines [28], depending of the O-PS engineered into the system.

Given that the O-PS section of the LPS is one of the most immunodominant STEC antigens [29, 30], we decided to explore a combined immunization strategy with adjuvated-bacteria followed by a booster with bacterial engineered O-PS-protein conjugates for the production of hybridomas secreting mAbs targeting O157 or O145 O-PS. This approach led to a selective proliferation of B-cell clones specific to O157 or O145 antigens, and probably enhancing the affinity of the secreted mAbs against the respective O-PS. These mAbs present an exceptional degree of specificity in the detection and identification of *E*. *coli* O157 and O145 priority serogroups, and may be of significant value for the development of improved rapid point-of-care-deployable assays for the detection of these STEC serogroups in food products, as well as in clinical and veterinary samples.

## Materials and methods

### Bacterial strains and culture conditions

The strains used in this work are listed in Table 1. These strains were grown on LB medium at 37°C for 18 to 20 h. Bacterial cells were harvested by centrifugation (5 min, 5000 rpm, 4°C), resuspended in PBS at approximately 10^9^ CFU/ml and heat-killed by incubation at 80°C for 30 min. All the STEC analyzed in this study came from a previously characterized serum collection provided by the Servicio Fisiopatogenia, Instituto Nacional de Enfermedades Infecciosas (INEI)-ANLIS Dr. Carlos G. Malbrán, the national reference laboratory (NRL) for HUS and diarrhea disease associated with diarrheagenic *E*.*coli*.

**Table 1:** Strains used in this work.

Strain	Reference or Source
***E*. *coli* strains**	
DH5α	Invitrogen
Serogroup O157:H7	INEI-ANLIS
Serogroup O145:NM	INEI-ANLIS
Serogroup O121:H19	INEI-ANLIS
Serogroup O111:NM	INEI-ANLIS
Serogroup O104:H4	INEI-ANLIS
Serogroup O103:H2	INEI-ANLIS
Serogroup O45:H2	INEI-ANLIS
Serogroup O26:H11	INEI-ANLIS
***B*. *abortus* 2308**	[31]
***S*. Urbana**	[32]
***Y*. *enterocolitica* O9**	[20]

### Production of recombinant glycoproteins

The O-PS protein conjugates were produced by exploiting the N-glycosylation pathway of *C*. *jejuni* in nonpathogenic bacteria. Briefly, the coexpression of the complete *C*. *jejuni pgl* locus and AcrA, a periplasmic component of a multidrug efflux pump that works as the aceptor protein, results in N-glycosylated AcrA in *E*. *coli* [33]. Recombinant O157-AcrA and O145-AcrA glycoproteins were produced and purified essentially as described by Melli et al [23]. Briefly, the nonpathogenic *E*. *coli* strains CLM24 AcrA-O157 and CLM24 AcrA-O145 (containing plasmids encoding *C*. *jejuni* OTase PglB and AcrA (carrier protein), and the *E*. *coli* O157 or O145 gene clusters, respectively), were induced by the addition of arabinose and isopropyl β-D-thiogalactopyranoside (IPTG). AcrA-glyconconjugates were purified from periplasmic fractions by Ni2+ affinity chromatography.

### Immunizations and hybridoma generation

All mice were obtained from our own breeding facility and were housed under specific pathogen free (SPF) conditions. For O157 immunizations we used seven 8- to 9-week-old male BALB/c mice, which were immunized intraperitoneally with 2x10^7^, 5x10^7^ and 1x10^8^ CFU of heat-killed *E*. *coli* O157 in incomplete Freund's adjuvant on days 0, 29 and 44 respectively. On day 50 we performed a test bleed, which was followed by a booster of 5x10^7^ CFU of heat-killed *E*. *coli* O157 in incomplete Freund's adjuvant on day 56. On days 62, 63 and 64 mouse #6 was administered 10 μg of O157-AcrA intraperitoneally. On day 65, mice were euthanized by cardiac puncture exsanguination under complete anesthesia, and the spleen of mouse #6 was used as a source of splenocytes for hybridomas development. For O145 immunizations we used six 8- to 9-week-old male BALB/c mice, which were immunized intraperitoneally with 5x10^7^, 1x10^8^, 2x10^8^ and 2x10^8^ CFU of heat-killed *E*. *coli* O145 in incomplete Freund's adjuvant on days 0, 21, 42 and 63 respectively. On day 70 a test bleed was performed, which was followed by the administration of 10 μg of O145-AcrA intraperitoneally to mouse #2 on days 84, 85 and 86. On day 87, mice were euthanized by cardiac puncture exsanguination under complete anesthesia, and the spleen of mouse #2 was used as a source of splenocytes for hybridomas development. Mice were monitored daily on weekdays. There were no deaths associated to immunizations or care. Serum samples from O157 mouse #6 and O145 mouse #2 were reserved as positive controls for agglutination assays. Hybridomas were produced by fusion of spleen cells with Sp2/0-Ag14 myeloma cells as described previously [34]. Screening of positive secreting hybridomas was carried out by glyco-iELISAs and the selected hybridomas were cloned twice to ensure single-cell cloning and the stability of the hybridomas. We have made two rounds of cloning by limiting dilution. The first time, we plated 1.5 cells/well in a 96 well plate. The second time, we plated 0.9 cells/well in a 96 well plate. After the second round of cloning, all wells were determined to be positive. A test sample was considered positive if the ratio (T/C) of the OD value in the test well (T) to that of the negative control well (C) was ≥2.1.

### Hybridoma supernatant concentration

Hybridoma supernatants were concentrated ∼5 times by ultrafiltration using a Molecular/Por Stirred Cell Ultra Filtration device (Spectrum) with a 50 KDa MWCO Molecular/Por ultrafiltration disc membrane (Spectrum).

### Glycoprotein indirect-enzyme-linked immunosorbent assay

Glyco-iELISA was performed as described previously [23], with minor modifications. Briefly, microtiter plates (Thermo Scientific Pierce 96-well polystyrene plates) were coated with 100 μl of AcrA, O157-AcrA or O145-AcrA (125 ng/well) in coating buffer (0.05 M carbonate buffer pH 9.6) for 18 h at 4°C. The plates were blocked in blocking buffer (5% bovine skim milk in TBS) for 1 h at 37°C and subsequently incubated with the indicated hybridoma supernatant or mice sera dilution for 1 h at RT in blocking buffer. Following four washing steps in TBS Tween-20 0.05%, plates were further incubated for 1 h at RT with HRP goat anti-mouse IgG secondary antibody (Sigma-Aldrich) at a 1:6000 dilution in blocking buffer. Finally, plates were washed four times in TBS 0.05% Tween-20 and after incubation with the substrate (0.3% H_2_O_2_, 0.1% 3,3',5,5'- tetramethylbenzidine [TMB] in 0.1 M citric acid pH 5) for 5 to 20 min at RT, the reaction was stopped with 0.2 M H_2_SO_4_. The absorbance at 450 nm was measured with a FilterMax F5 Multi-Mode microplate reader (Molecular Devices).

### Isotyping of immunoglobulins

The isotypes of the mAbs were determined with the Mouse Ig Isotyping Ready-SET-Go kit (Affymetrix, eBioscience) according to the manufacturer's instructions.

### Indirect enzyme-linked immunosorbent assay

Microtiter plates (Thermo Scientific Pierce 96-well polystyrene plates) were coated with 100 μl of heat-killed bacteria (∼10^7^ CFU/well) in coating buffer (0.05 M carbonate buffer pH 9.6) for 18 h at 4°C. Following incubation in blocking buffer (5% bovine skim milk in TBS) for 1 h at 37°C, the plates were further incubated with 1:100 of the indicated hybridoma supernatant concentrate or with 1:2000 mouse anti-*Brucella* O-PS (M84) mAb [35] for 1 h at RT in blocking buffer. Detection of antibodies with secondary antibody, reaction development and absorbance measurement were carried out as described in *glycoprotein indirect-enzyme-linked immunosorbent assay*.

### Western blotting

Bacterial cell lysates, glycoproteins and non-glycosylated AcrA were resolved on 10% SDS-PAGE. After transfer to a nitrocellulose membrane (Hybond-ECL, GE Healthcare), analysis by immunoblotting was performed using 1:200 O157 1E10, 1:1000 O157 3F10, 1:150 O157 10G2, 1:100 O145 2H6, 1:1000 O145 4C8 or 1:100 O145 4E6 hybridoma supernatant concentrates. Bound mAbs were recognized with Alexa Fluor 680 goat anti-mouse IgG secondary antibody (Invitrogen) at a 1:20000 dilution and visualized with an Oddysey Infrared Imager (Li-Cor).

### Flow cytometry

Heat-killed bacteria (∼10^6^ CFU) were blocked with 3% BSA in TBS for 1 h at RT and washed twice in TBS previous to surface staining with the indicated hybridoma supernatant concentrate for 1 h at RT. Following two washing steps in TBS, bacteria were incubated for 1 h at RT with Alexa Fluor 488 goat anti-mouse IgG secondary antibody (Invitrogen) at a 1:500 dilution in blocking buffer. Finally, bacteria were washed twice in TBS and samples were measured with a CyFlow Space cytometer (Partec). Data analysis was performed with WinMDI 2.9 software.

### Confocal microscopy

*E*. *coli* O157:H7 and O145:NM strains were plated on LB agar plates and incubated overnight at 37°C. A group of colonies were resuspended in PBS and fixed with 4% paraformaldehyde for 20 min at RT. After fixation, bacteria were washed with PBS and incubated with the indicated hybridoma supernatant concentrate for 1 h at RT. Following three washing steps in PBS, bacteria were incubated for 1 h at RT with Alexa Fluor 488 goat anti-mouse IgG secondary antibody (Invitrogen) at a 1:2000 dilution in PBS. Bacteria were washed three times in PBS and placed on a microscope slide that was layered with a 1% agarose pad in PBS as previously described [36]. Image acquisition was performed with a IX81 microscope with an Olympus FV1000 confocal module attached, using a 60X PLAPO objective and 1.42 NA. At least three fields of each stain were randomly selected for analysis. Images were processed with the NIH Image J software.

### Agglutination assay

Heat killed bacteria (∼10^5^ CFU) were incubated in round bottom microtiter plates (Corning Costar 96-well round bottom cell culture plates) in a final volume of 120 μl with the indicated hybridoma supernatant concentrate or 1:10 mouse antisera in 0.1% crystal violet dye for 30 min at 37°C, and were further refrigerated overnight at 4°C.

### Statistical analysis

The software GraphPad Prism 5.0 (GraphPad Software, La Jolla, CA, USA) was used for the non linear fitting of the standard curves to a 4 parameter logistic regression and for the calculation of the IC50 parameter.

### Ethics statement

The protocol of animal immunization followed in this study was approved by the Committee on the Ethics of Animal Experiments of the Universidad Nacional de San Martín, according to the recommendations of the Guide for the Care and Use of Laboratory Animals of the National Institutes of Health.

## Results

### Development of highly specific monoclonal antibodies to O157 and O145 serogroups

**Fig 1:**
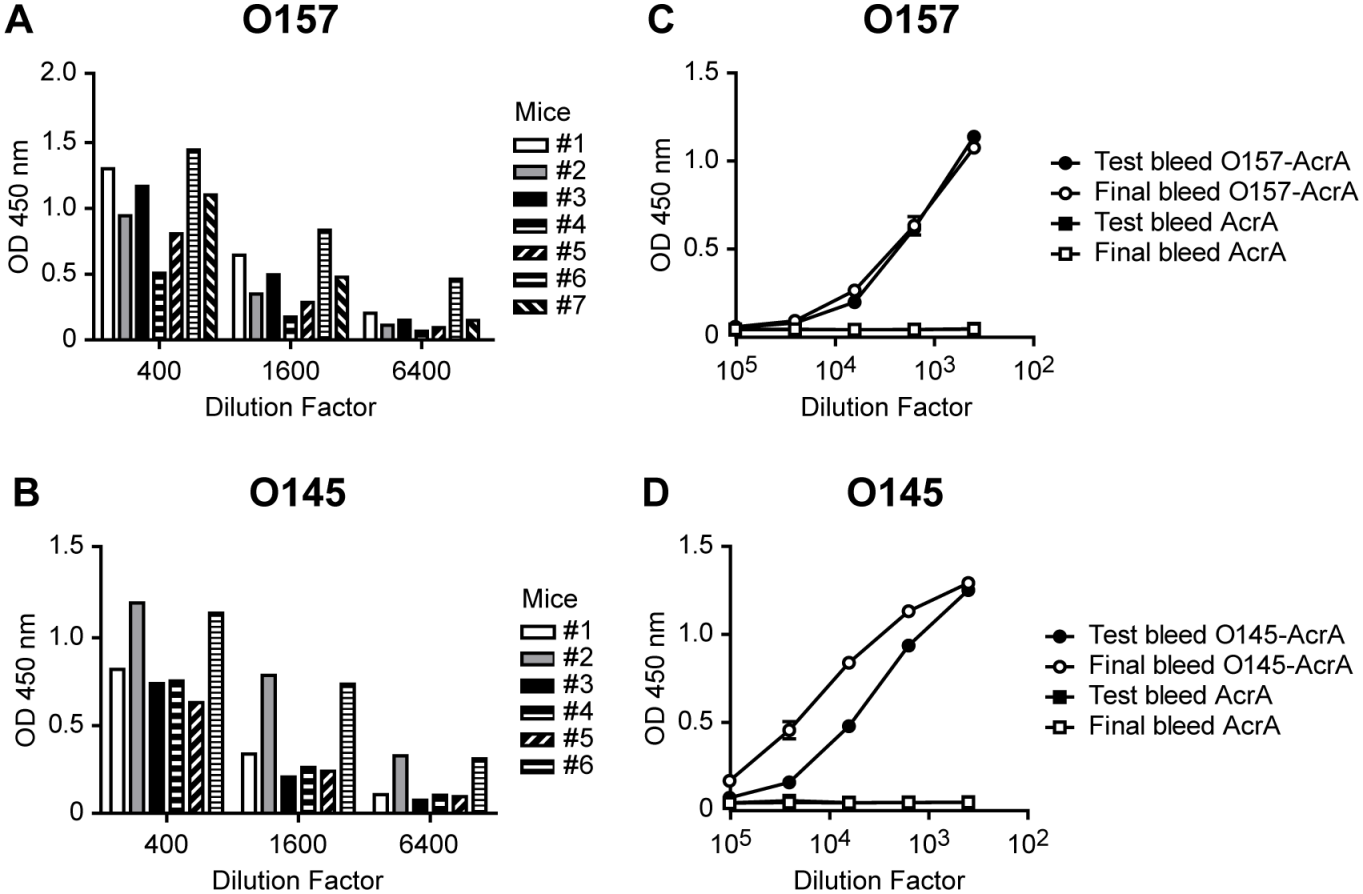
Development of hybridomas secreting mAbs against *E*. *coli* O157 and O145. **(A-B)** Analysis of test bleed sera titers by glyco-iELISA of the mice immunized with heat-killed *E*. *coli* O157 **(A)** or *E*. *coli* O145 **(B)** strains to select the mouse destined to fusion and generation of hybridomas. **(C-D)** Comparison of test bleed and final bleed sera titers of the mice used for fusion by O157-AcrA **(C)** or O145-AcrA **(D)** glyco-iELISAs. In **(C)** and **(D)** data represents mean±SD of two sample replicates.

The success in the development of mAbs largely depends on the ability to induce a strong humoral response on the donor of the splenocytes used to produce hybridomas. After testing the IgG response of mice immunized intraperitoneally with heat-killed *E*. *coli* O157 or O145 bacteria cells (Fig 1A and 1B), the best responders on a glycoprotein indirect-enzyme-linked immunosorbent assay (glyco-iELISA) were further immunized with purified O157-AcrA or O145-AcrA glycoprotein conjugates, respectively. The purpose of these immunizations was to induce the selective proliferation of O-PS specific B-cell clones during a short period of time that would not allow an IgG response against the AcrA carrier protein. Previous immunizations with O-PS-AcrA conjugates only gave place to hybridoma populations secreting mAbs to AcrA (not shown), thus evidencing the potent antigenicity of this carrier protein. To assess whether these immunizations with soluble glycoproteins raised the response towards the LPS O-PS antigen, we performed glyco-iELISAs to compare the test bleed and final bleed sera titers of the mice sacrificed for fusion (Fig 1C and 1D). We observed a 33% titer increase at a 1:6400 sera dilution and a 184% titer increase at a 1:25600 sera dilution after the immunization with O157-AcrA and O145-AcrA, respectively (S1 Table). This suggests that the final boosts with bacterial engineered O-PS-protein conjugates led to a selective proliferation of B-cell clones specific to O157 or O145 antigens, probably enhancing the affinity of the secreted mAbs against the respective O-PS. We obtained 18,8% and 13,3% of positive and specific hybridoma populations to O157 and O145, respectively. As expected, we only obtained four and none hybridoma populations against AcrA in the O157 and O145 selected populations, thus validating our combined approach for immunizations consisting of heat-killed bacteria followed by soluble O-PS-protein conjugates. After two rounds of cloning, we selected three specific hybridoma clones for O157 (1E10, 3F10 and 10G2) and three for O145 (2H6, 4C8 and 4E6). We determined that the isotypes of the mAbs were IgG1 for O145 4C8 and O145 4E6, IgG2b for O157 3F10 and IgG3 for O157 1E10, O157 10G2 and O145 2H6, all of them containing a lambda light chain (S2 Table).

### Specificity of O157 and O145 monoclonal antibodies

**Fig 2:**
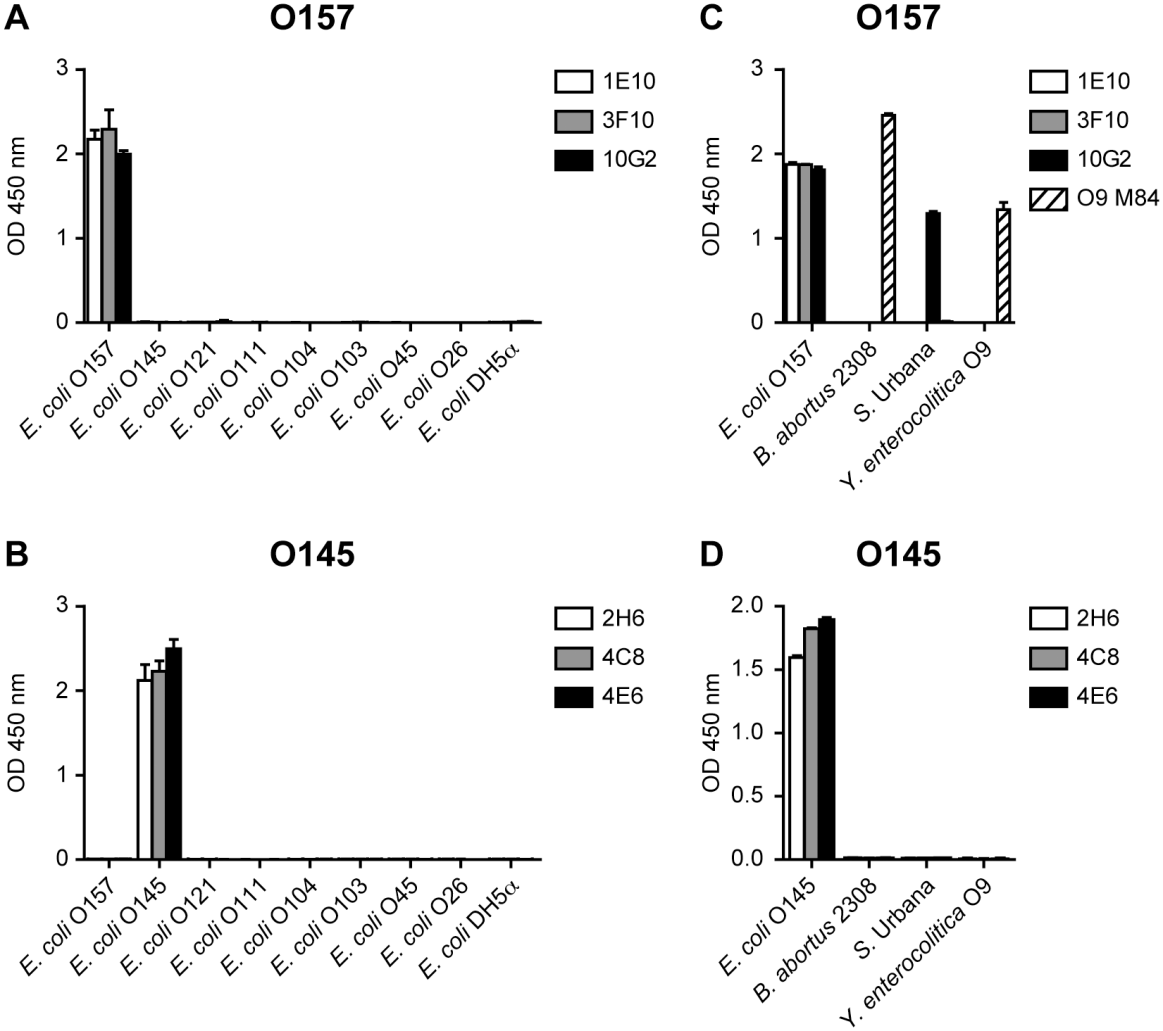
Specificity of O157 and O145 mAbs towards STEC strains by iELISA. iELISA of representative STEC strains of serogroups O157, O145, O121, O111, O104, O103, O45 and O26 **(A-B)**, and iELISA of *B*. *abortus* 2308, *S*. Urbana, *Y*. *enterocolitica* O9 and *E*. *coli* O157 **(C)** or *E*. *coli* O145 **(D)**, using O157 1E10, 3F10 and 10G2 mAbs **(A,C)** or O145 2H6, 4C8 and 4E6 mAbs **(B,D)**. In **(A-B)** non-pathogenic *E*. *coli* DH5α strain was used as a negative control, and in **(C)** O9 M84 mAb was used as *B*. *abortus* and *Y*. *enterocolitica* O9 positive control. Each point of the curve represents the mean±SD of three sample replicates.

**Fig 3:**
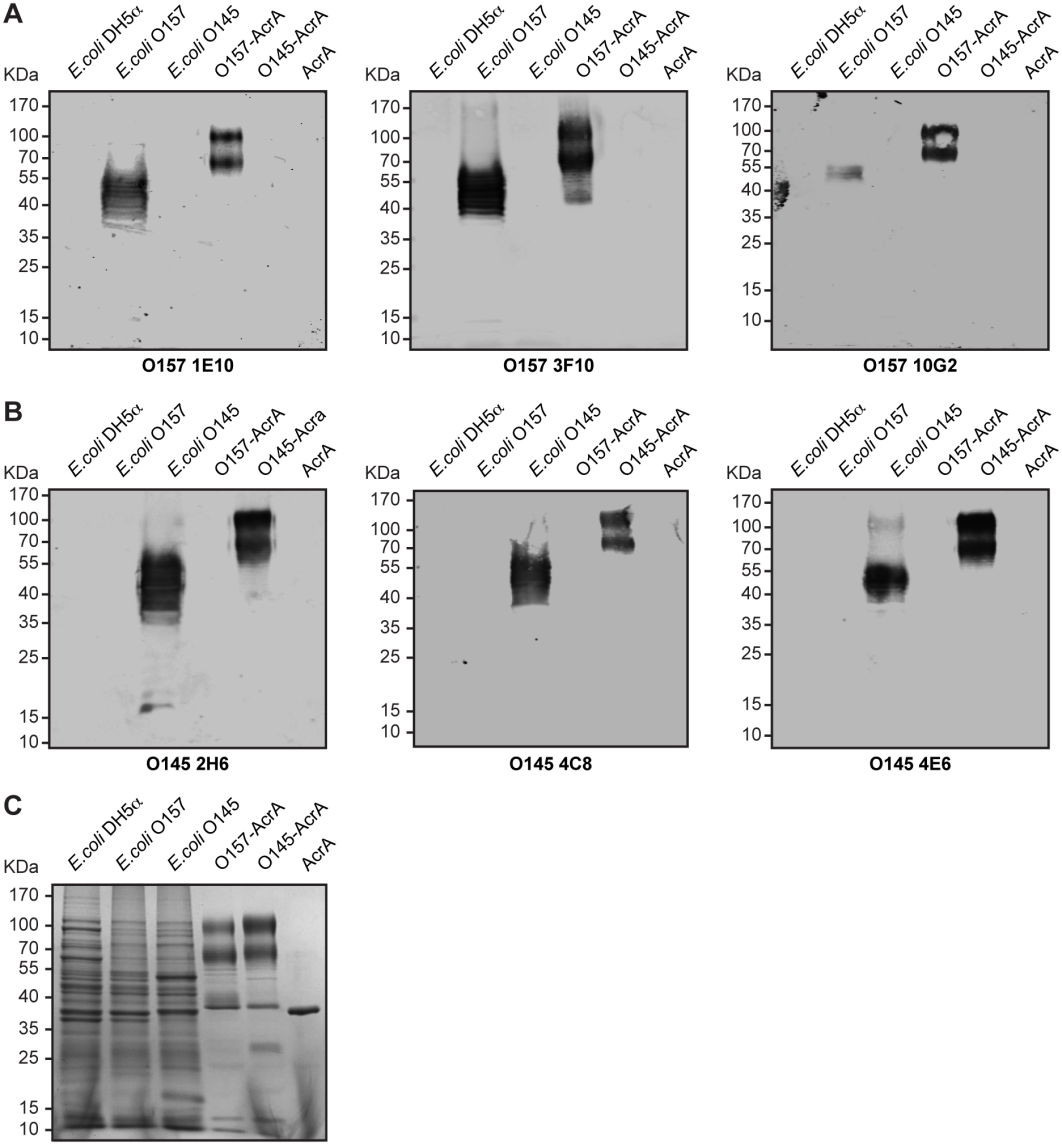
Immunoblot analysis of *E*. *coli* strains and recombinant glycoproteins with O157 and O145 mAbs. Immunoblot using mAbs O157 1E10, 3F10 and 10G2 **(A)** or mAbs O145 2H6, 4C8 and 4E6 **(B)**. Non-pathogenic *E*. *coli* DH5α strain and non-glycosylated AcrA were loaded as controls. SDS-PAGE analysis of *E*.*coli* O157 and O145 strains, and purified O157-AcrA and O145-AcrA glycoproteins by Coomassie brilliant blue staining **(C).** The positions of the molecular mass standards are indicated on the left.

To test the specificity of the developed mAbs, we analyzed their reactivity by iELISA against members of the “big six” STEC serogroups (O121, O111, O103, O45 and O26), plus the serogroup O104. No cross-reactivity was observed for neither mAb towards the other six STEC priority serogroups nor to a non-pathogenic *E*. *coli* DH5α strain (Fig 2A and 2B). It has been proposed that the serological cross-reactions observed between *E*. *coli* O157 and other bacteria, such as those from the *Salmonella* group N [37], *Brucella abortus* [38] and *Yersinia enterocolitica* O9 [39, 40], are due to the presence of a common structural epitope consisting of N-acetyl derivatives of 1,2-linked 4-amino-4,6-dideoxy-α-D-mannopyranosyl residues contained in the O-PS repeating unit of their LPS [41]. Of the mAbs characterized here, 1E10 and 3F10 did not show any cross-reactivity towards *B*. *abortus* 2308, *Salmonella* serovar Urbana or *Y*. *enterocolitica* O9 whole cells, while 10G2 mAb only partially cross-reacted with *S*. Urbana (Fig 2C). As expected, none of the developed O145 mAbs cross-reacted with *B*. *abortus* 2308, *S*. Urbana or *Y*. *enterocolitica* O9 (Fig 2D), given that *E*. *coli* O145 does not share common structural epitopes in the O-PS repeating unit of its LPS with these enterobacteria [42]. Taken together, these results suggest that the O157 and O145 mAbs are highly specific to the corresponding O-PS and do not present cross-reactions with the O antigen of other STEC priority serogroups or other enterobacteria that are associated with similar clinical manifestations. To further confirm the specificity of the obtained mAbs, we evaluated their reactivity against the corresponding LPS fraction in bacterial cell lysates and recombinant glycoproteins by Western blot (Fig 3). Immunoblot analysis showed that the O157 and O145 mAbs recognize the characteristic LPS fraction present in the corresponding STEC strain (Fig 3A and 3B), which is typically detected as a band ladder due to the repeated saccharide units. We could not detect cross-reactivity of O157 or O145 mAbs against a non-pathogenic *E*. *coli* DH5α strain or against the non-targeted STEC strain (Fig 3A and 3B). All mAbs recognized the corresponding O157-AcrA or O145-AcrA glycoproteins, but did not show reactivity against the non-glycosylated AcrA nor against the non-targeted glycoprotein (Fig 3A and 3B). Coomassie brilliant blue staining of SDS-PAGE showed the band patterns of a non-pathogenic *E*. *coli* DH5α, *E*. *coli* O157 and O145 lysates, and the characteristic O157-AcrA and O145-AcrA clusters of bands at ∼70 and ∼100 KDa [23] (Fig 3C). The presence of a band of lower molecular weight (∼38 KDa) in O157-AcrA and O145-AcrA lanes corresponds to the non-glycosylated AcrA form (Fig 3C). These results indicate that the developed mAbs specifically recognize the O-PS moiety of the corresponding LPS, thus validating the use of O-PS-AcrA glycoconjugates for the detection of specific antibodies.

### Serotyping of *E*. *coli* O157 and O145 with specific monoclonal antibodies

**Fig 4:**
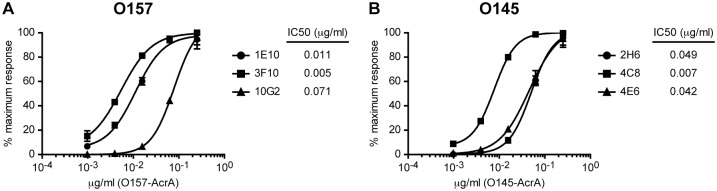
Relative affinity of mAbs towards O157-AcrA and O145-AcrA by glyco-iELISA. Standard curves of O157-AcrA **(A)** or O145-AcrA **(B)** detected by glyco-iELISA with the use of mAbs O157 1E10, 3F10 and 10G2 (3:8 hybridoma supernatant dilution) **(A)** or mAbs O145 2H6, 4C8 and 4E6 (1:2 hybridoma supernatant dilution) **(B)**. Each point of the curve represents the mean±SD of two sample replicates. IC50 values of the mAbs are indicated.

In order to characterize the selected mAbs, we studied their relative affinity for the corresponding glycoprotein. For this, we immobilized different concentrations of O157-AcrA or O145-AcrA and detected them with the mAbs by glyco-iELISA. The relative affinity of each mAb for the antigen was calculated as the concentration of the antigen conferring a 50% reduction of the peak signal in the ELISA (IC50). The sensitivity of O157 3F10 mAb was about fourteen or two times higher than that of O157 10G2 or O157 1E10 mAbs, respectively (Fig 4A). The relative affinity of O145 4C8 was six or seven times higher than that of O145 4E6 or O145 2H6 mAbs, respectively (Fig 4B).

**Fig 5:**
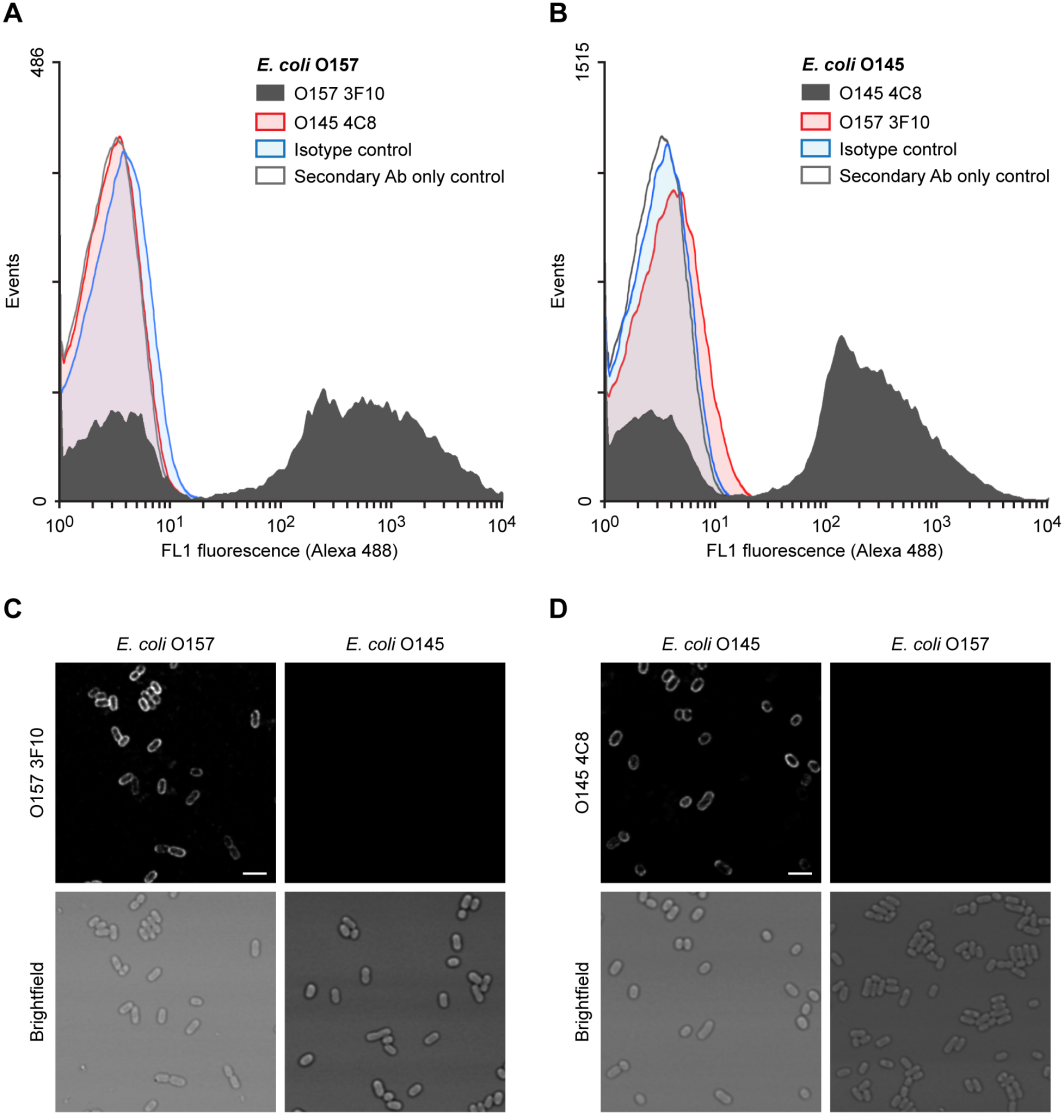
Surface staining of O157 and O145 STEC strains with O157 and O145 mAbs. **(A-B)** Binding of O157 3F10 and O145 4C8 mAbs to *E*.*coli* O157 **(A)** or *E*.*coli* O145 **(B)** heat-killed bacteria assessed by flow cytometry. An irrelevant isotype-matched murine mAb or only secondary Ab were used as controls. **(C-D)** Immunostaining of *E*.*coli* O157 and O145 with O157 3F10 **(C)** and O145 4C8 **(D)** mAbs, visualized by confocal microscopy. Scale bar, 3 μm.

**Fig 6:**
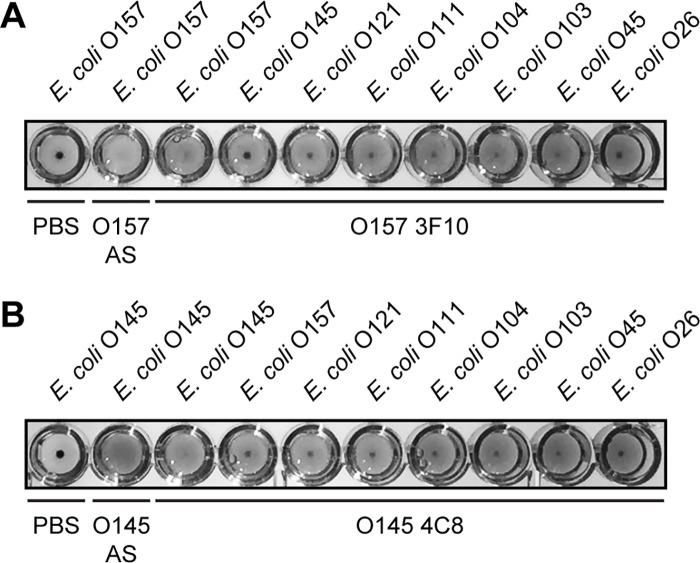
Agglutination assay of STEC strains with O157 and O145 mAbs. Detection of O157 **(A)** and O145 **(B)** antigens in representative STEC strains of serogroups O157, O145, O121, O111, O104, O103, O45 and O26 by agglutination assays using O157 3F10 mAb or O157 mouse antisera **(A)**, or O145 4C8 mAb or O145 mouse antisera **(B)**. AS: antisera.

The ability of these mAbs to bind the surface of whole bacteria cells, which has a potential value for serotyping and diagnosis, was evaluated by flow cytometry assays. This technique has been proposed as a possible alternative to current assays for the detection of *E*. *coli* O157 due to the possibility for automation and rapid detection [43, 44]. MAbs O157 3F10 or O145 4C8 were able to bind *E*. *coli* O157 or *E*. *coli* O145 strains respectively, while no cross-reactivity was observed (Fig 5A and 5B). Sensitivity was enough to specifically detect the presence of *E*. *coli* in suspension, suggesting that this approach can also be used for diluted samples. This result shows that the recognized epitope on the O-PS from O157 or O145 serogroups is accessible on the surface of the bacteria, providing the basis for detection strategies involving live cells. To confirm the interaction of O157 3F10 and O145 4C8 mAbs with the O-PS on the bacterial surface, we stained bacteria with the corresponding mAb and analyzed O-PS localization by confocal microscopy (Fig 5C and 5D). As expected, we observed the typical staining of *E*. *coli* surface with the specific mAb, and no cross-reactivity was observed (Fig 5C and 5D). It is important to point out that all bacteria in the sample were stained by the specific mAb, showing the sensitivity of this direct strategy. These results add further validation to the specificity and sensitivity of these mAbs for the detection of pathogenic *E*. *coli* in suspension. To continue exploring the diagnostic potential of the developed mAbs, we assessed their ability to agglutinate representative STEC strains of serogroups O157, O145, O121, O111, O104, O103, O45 and O26. In this assay, heat-killed *E*. *coli* O157 or O145 whole bacteria incubated in PBS do not agglutinate, showing a characteristic dot in the bottom of U-shaped 96-well plates (Fig 6). Incubation of the same bacteria with O157 or O145 specific polyclonal mouse antisera gave positive agglutination reactions, as expected (Fig 6). Testing of O157 3F10 or O145 4C8 hybridoma supernatants with the target bacterial serogroup also gave positive agglutination reactions (Fig 6). However, we did not observe agglutination when testing the hybridoma supernatants with non-specific serogroups. Taken together, these results further confirm the serogroup specificity of the developed mAbs and demonstrate their accessibility to the recognized epitope in the bacterial surface, either by flow cytometry or immunofluorescence staining of the surface of the cells, or by allowing the agglutination of bacteria in the presence of the specific mAb. In addition, this thorough characterization unveil the applicability of O157 and O145 mAbs as reliable tools in routine serotyping and diagnostic assays, making these molecules ideal to differentiate *E*. *coli* O157 and O145 serogroups.

## Discussion

The implementation of primary prevention strategies in public health are essential to decrease the morbidity and mortality associated with HUS, as it is emphasized by the World Health Organization. These should be accompanied by the promotion of educational programs for the population, warning them about the risks of STEC as well as its transmission routes and prevention strategies [11]. Additionally, there is an imperative need to incorporate rapid, sensitive and accurate technologies to detect and characterize pathogenic STEC strains along the food chain to ensure food safety. Argentina shows the highest rate of HUS worldwide, with 400 new cases annually, which is ten times higher than in developed countries [11]. For these reasons, the present investigation aimed at developing highly specific mAbs against the O-PS section of the LPS of the dominant STEC-associated HUS serogroups in Argentina: O157 and O145.

Current development of mAbs against LPS moieties from pathogenic bacteria rely on the use of purified LPS as an antigen for immunization or screening [45, 46], while the use of whole bacteria imply larger screening sets of mAb secreting cells [47]. Both strategies involve the culture of variable amounts of the pathogenic bacteria, assuming an important hazard for the operator. With this in mind, we used a glyco-engineered recombinant protein-conjugate as antigen, which is synthesized in non-pathogenic bacteria, allowing the production of larger quantities of antigen under biosecure conditions [23]. Using this strategy we developed specific mAbs to the O antigen of the LPS of STEC O157 and O145 strains. The production of hybridomas secreting O157 or O145 mAbs was carried out through a combined immunization strategy with adjuvated-bacterial immunizations, which typically provide a robust B cell response. To provide specificity by promoting proliferation of specific O-PS specific B cells, which are used to produce hybridomas, mice were further immunized with soluble glycoengineered recombinant O-PS-protein conjugates O157-AcrA and O145-AcrA [23]. These recombinant glycoproteins have been efficiently used to discriminate between STEC O157- and O145-infected patients, as well as to diagnose HUS [23]. It is well known that the O-PS section of the LPS is one of the most immunodominant STEC antigens [29, 30]. Specificity of the developed mAbs largely depends on cross-reactivity with common epitopes shared by STEC serotypes and other bacteria, such as the core polysaccharide or lipid A of the LPS, which are absent in AcrA glycoconjugates. These immunizations were close enough to the day of spleen removal in order to prevent an IgG response to the carrier protein of the recombinant O-PS. As expected, we observed a titer increase in mice as a consequence of immunization with the soluble O-PS-protein conjugate, indicating that the final boosts with the glycoproteins led to a selective proliferation of B-cell clones specific to O157 or O145 antigens (Fig 1), and also probably to an enhanced affinity of the secreted antibodies against the respective O-PS. Besides, it should be noted that the screening of positive mAb-producing hybridomas was accomplished by glyco-iELISAs, thereby ensuring the selection of specific mAb-secreting hybridomas for cloning. The generation of hybridomas with the O-PS-protein conjugate immunization strategy and their screening and selection through a glyco-iELISA approach might explain the high yield of the obtained hybridoma populations secreting specific mAbs against O157 and O145. We observed 18,8% and 13,3% of positive and specific hybridoma populations to O157 and O145 respectively (Fig 1). These fusion efficiencies are higher than the ones usually obtained by conventional subcutaneous immunization and intraperitoneal boost followed by splenic lymphocyte fusion, which are, in the best case, not superior than 8% [48, 49].

Binding of mAbs to O157 and O145 STEC whole bacteria by iELISA further demonstrated their specificity, showing no recognition of STEC strains from serogroups O121, O111, O104, O103, O45, O26. Western blot assays showed that O157 and O145 mAbs detected the characteristic LPS ladders containing several O side chain repeat units in the corresponding STEC strain, as well as the O157-AcrA and O145-AcrA glycoproteins respectively, without cross-reacting with non-glycosylated AcrA. We assume that the specificity of the mAbs could be explained by the O-PS-protein conjugation approach used to generate the hybridomas since only the O-PS, the outer moiety of the LPS, is coupled to the carrier protein. Thus, this strategy prevented any cross-reactions with other antigens and/or common epitopes shared by STEC serotypes and other bacteria, such as the core polysaccharide or lipid A of the LPS. In particular, developing mAbs specific to *E*. *coli* O157:H7 constitutes a challenge because this serotype shares a structural epitope in its LPS with other bacteria−including *Salmonella* group N, *B*. *abortus* and *Y*. *enterocolitica* O9−, and this epitope is responsible for the frequently observed serological cross-reactions between them [37–41, 50]. Our iELISA results demonstrated that O157 1E10 and 3F10 mAbs did not recognize any epitope in *B*. *abortus* 2308, *S*. Urbana or *Y*. *enterocolitica* O9 O-PS antigens, while 10G2 mAb only partially cross-reacted with *S*. Urbana. Therefore, we show that highly specific mAbs against the O-PS moiety of O157 STEC can be efficiently made with this approach, having a very good potential to discriminate *E*. *coli* O157:H7 from other human, veterinary and foodborne pathogens that are associated with similar clinical manifestations. *E*. *coli* O157 O-PS antigen is an unbranched linear polisaccharide with a tetrasaccharide repeting unit, which contains a 1,2-linked 4-amino-4,6-dideoxy-α-D-mannopyranosyl (D-perosamine) residue [41]. The LPS of *S*. Urbana, that belongs to the *Salmonella* O30_1_O30_2_ subgroup of the *Salmonella* Kauffmann-White group N, is a repeating pentasaccharide unit composed of a tetrasaccharide related to the tetrasaccharide of *E*. *coli* O157, with an additional hexose residue at a branch point in the pentasaccharide repeating unit [32]. Finally, the O-PS antigens of *B*. *abortus* and *Y*. *enterocolitica* O9 are structurally identical, characterized as linear homopolymers of D-perosamine units [51, 52]. Considering the structures of the O-PS antigens of these enterobacteria, and since none of the three developed mAbs reacted with *B*. *abortus* 2308 or *Y*. *entercolitica* O9, we can conclude that they do not recognize the shared D-perosamine residue in the *E*. *coli* O157 O-PS antigen. Moreover, we propose that the specificity of the epitopes of 1E10 and 3F10 mAbs, which did not cross-react with *S*. Urbana, is at or proximal to the additional hexose substitution site, and that the additional hexose in the pentasaccharide repeating unit of *S*. Urbana blocks or sterically hinders the binding of 1E10 and 3F10 mAbs to the O-chains of its LPS. In addition, the fact that the 10G2 mAb partially cross-reacted with *S*. Urbana suggests that its epitope is distal to the substitution site and that its specificity is different from those of 1E10 and 3F10 mAbs. It is interesting to emphasize that we have developed for the first time two mAbs against *E*. *coli* O157 O-PS antigen, 1E10 and 3F10, which do not present any cross-reactions with *B*. *abortus*, *S*. Urbana or *Y*. *enterocolitica* O9 LPS O-chains. In previous work by Westerman et al the development of mAbs towards the O-PS of *E*. *coli* O157 was described, but they found them to cross-react with *S*. Urbana [53], while the mAbs developed by Guttikonda et al. were not analyzed for cross-reactions to *B*. *abortus* or *Y*. *entercolitica* O9 [54].

The main route of transmission of O157 and non-O157 STEC is contaminated food products, such as ground beef, raw or undercooked meat, burgers, sausages, unpasteurized milk, yogurt, cheese, mayonnaise, potatoes, lettuce, bean and alfalfa sprouts, unpasteurized apple cider and water, among others [55–58]. Food contamination is mainly due to contact with cattle feces. In Argentina, non-O157 STEC was detected in 8.4% of frozen burgers and STEC O157 in 3.9% of retail meat products [59, 60]. Other modes of transmission include direct contact with animals, cross-contamination during food preparation and person-to-person transmission by fecal-oral route [5, 6]. It is important to note that the infective dose capable of causing disease by STEC strains is 10 to 100 bacteria per gram of food [11]. Driven by the need of primary prevention strategies that comprise rapid, sensitive and specific technologies to detect pathogenic O157 and O145 STEC strains in food manufacturing facilities, we tested the ability of the developed mAbs to bind the surface of whole bacteria cells in serotyping assays. We confirmed that O157 3F10 and O145 4C8 mAbs, the ones with the highest affinity for the corresponding O-PS, are reliable reagents to specifically detect O157 and O145 STEC strains respectively by flow cytometry. The specific detection of *E*. *coli* O157 and O145 in suspension not only would demonstrate the presence of the pathogen, but also could be used for quality control by the selective enrichment of live bacteria by fluorescence-activated cell sorting (FACS) from food or environmental samples containing mixed bacterial populations [44, 61]. This would allow quantitation of bacterial burden, while enrichment of the sample would provide a reduction on time compared with plate-counting methods [62]. Furthermore, these O157 3F10 and O145 4C8 mAbs showed that they can be used as clinical diagnosis tools to agglutinate specific STEC serogroups, thus providing a rapid method to discriminate not only between *E*. *coli* O157 and O145, but also between *E*. *coli* O157 and enterobacteria such as *Salmonella* group N and *Y*. *enterocolitica* O9.

In the last years, a "reproducibility crisis" phenomenon has emerged as a consequence of flaws in the reliability of antibodies [63, 64]. In this context, it is important to emphasize that O157 and O145 mAbs have been carefully validated against their antigens, and against antigens with the higher chances of cross-reaction, providing the basis for reproducibility in clinical diagnosis and food quality control in manufacturing facilities.

## Supporting information

S1 Table Test bleed and final bleed absolute and relative OD 450 nm values of the mice used for fusion assessed by O157-AcrA or O145-AcrA glyco-iELISA. (PDF)

S2 Table Isotypes of O157 and O145 secreting hybridomas. (PDF)

S1 Checklist The ARRIVE guidelines checklist. (PDF)
