# Supplementary material for: A recombinant O-polysaccharide-protein conjugate approach to develop highly specific monoclonal antibodies to Shiga toxin-producing Escherichia coli O157 and O145 serogroups
Source: PLoS One. 2017 Oct 5;12(10):e0182452. doi: 10.1371/journal.pone.0182452 (PMC5628784; doi:10.1371/journal.pone.0182452)
Supplement: S1 Table — (PDF) [file pone.0182452.s001.pdf]

	Dilution factor	OD 450 nm absolute values								Mean OD 450 nm relative values (%)	
O157		Test bleed O157-AcrA		Final bleed O157-AcrA		Test bleed AcrA		Final bleed AcrA		Test bleed O157-AcrA	Final bleed O157-AcrA
	400	1.16	1.12	1.08	1.08	0.05	0.05	0.05	0.05	100	94.5
	1600	0.63	0.60	0.60	0.67	0.04	0.04	0.04	0.04	100	102.4
	6400	0.20	0.20	0.27	0.27	0.04	0.04	0.05	0.04	100	133.4
	25600	0.08	0.08	0.09	0.10	0.05	0.04	0.05	0.04	100	116.1
	102400	0.05	0.06	0.06	0.06	0.04	0.04	0.05	0.05	100	109.2
O145		Test bleed O145-AcrA		Final bleed O145-AcrA		Test bleed AcrA		Final bleed AcrA		Test bleed O145-AcrA	Final bleed O145-AcrA
	400	1.25	1.25	1.28	1.31	0.05	0.05	0.05	0.05	100	103.2
	1600	0.93	0.94	1.12	1.14	0.05	0.05	0.05	0.05	100	120.8
	6400	0.48	0.48	0.84	0.84	0.05	0.05	0.05	0.04	100	175.0
	25600	0.17	0.15	0.49	0.42	0.05	0.07	0.05	0.04	100	283.5
	102400	0.07	0.08	0.17	0.16	0.04	0.04	0.04	0.04	100	229.9
