# Supplementary material for: A recombinant O-polysaccharide-protein conjugate approach to develop highly specific monoclonal antibodies to Shiga toxin-producing Escherichia coli O157 and O145 serogroups
Source: PLoS One. 2017 Oct 5;12(10):e0182452. doi: 10.1371/journal.pone.0182452 (PMC5628784; doi:10.1371/journal.pone.0182452)
Supplement: S2 Table — (PDF) [file pone.0182452.s002.pdf]

	O157			O145		
	**1E10**	**3F10**	**10G2**	**2H6**	**4C8**	**4E6**
**IgG1**	0.05	0.09	0.06	0.08	**3.05**	**3.17**
**IgG2a**	0.06	0.11	0.18	0.06	0.10	0.07
**IgG2b**	0.06	**3.38**	0.05	0.06	0.06	0.06
**IgG3**	**2.71**	0.10	**2.46**	**2.46**	0.06	0.06
**IgA**	0.05	0.09	0.05	0.05	0.05	0.05
**IgM**	0.06	0.08	0.06	0.06	0.07	0.06
**Lambda**	**1.49**	**3.08**	**1.04**	**1.41**	**2.10**	**2.38**
**Kappa**	0.08	0.10	0.10	0.08	0.10	0.08
